# The adipose tissue stromal vascular fraction secretome enhances the proliferation but inhibits the differentiation of myoblasts

**DOI:** 10.1186/s13287-018-1096-6

**Published:** 2018-12-20

**Authors:** R. El-Habta, M. Sloniecka, P. J. Kingham, L. J. Backman

**Affiliations:** Department of Integrative Medical Biology, Section for Anatomy, Umeå University, SE-901 87 Umeå, Sweden

**Keywords:** Adipose stem cells, Differentiation, HGF, Myoblasts, Myotubes, Myogenesis, Proliferation, SVF

## Abstract

**Background:**

Adipose tissue is an excellent source for isolation of stem cells for treating various clinical conditions including injuries to the neuromuscular system. Many previous studies have focused on differentiating these adipose stem cells (ASCs) towards a Schwann cell-like phenotype (dASCs), which can enhance axon regeneration and reduce muscle atrophy. However, the stromal vascular fraction (SVF), from which the ASCs are derived, also exerts broad regenerative potential and might provide a faster route to clinical translation of the cell therapies for treatment of neuromuscular disorders.

**Methods:**

The aim of this study was to establish the effects of SVF cells on the proliferation and differentiation of myoblasts using indirect co-culture experiments. A Growth Factor PCR Array was used to compare the secretomes of SVF and dASCs, and the downstream signaling pathways were investigated.

**Results:**

SVF cells, unlike culture-expanded dASCs, expressed and secreted hepatocyte growth factor (HGF) at concentrations sufficient to enhance the proliferation of myoblasts. Pharmacological inhibitor studies revealed that the signal is mediated via ERK1/2 phosphorylation and that the effect is significantly reduced by the addition of 100 pM Norleual, a specific HGF inhibitor. When myoblasts were differentiated into multinucleated myotubes, the SVF cells reduced the expression levels of fast-type myosin heavy chain (MyHC2) suggesting an inhibition of the differentiation process.

**Conclusions:**

In summary, this study shows the importance of HGF as a mediator of the SVF effects on myoblasts and provides further evidence for the importance of the secretome in cell therapy and regenerative medicine applications.

**Electronic supplementary material:**

The online version of this article (10.1186/s13287-018-1096-6) contains supplementary material, which is available to authorized users.

## Background

One of the biggest challenges of modern medicine is to repair and regenerate damaged tissue in the neuromusculoskeletal systems. Peripheral nerve injuries affect hundreds of thousands of people each year and lead to immediate loss of voluntary function and rapid loss of muscle mass [1]. With time, denervated skeletal muscle tissue is replaced by fibrous connective tissue and fat. In parallel, there is an activation of intrinsic satellite cells, which is a sign of neomyogenesis. However, despite this endogenous response, the absence of innervation ultimately leads to a point at which it is impossible to regain normal muscle function [2].

Mesenchymal stem cells (MSCs) have great clinical potential to treat many human diseases and regenerate damaged tissue due to their anti-inflammatory, anti-scarring, angiogenic, and pro-healing functions [3, 4]. Adipose tissue is an excellent source for isolation of MSCs [5], and previous studies have shown how adipose derived stem cells (ASCs) have pro-regenerative effects in the injured peripheral nervous system [6–8]. Furthermore, a study by Schaakxs et al. showed how ASCs differentiated towards a Schwann cell-like phenotype (dASCs) could attenuate muscle atrophy [9]. Recently, we reported that the dASCs enhanced the proliferation of myoblasts in vitro through paracrine secretion of acetylcholine [10]. Others have reported that dASCs could promote neurite outgrowth and form myelin structures with neuronal cells [11, 12]. However, to obtain the dASCs for clinical use requires that the cells are expanded and differentiated in vitro under GMP conditions which is time consuming and costly. In the current study, we therefore wanted to test a different approach: to use freshly isolated stromal vascular fraction (SVF) cells instead of culture expanded differentiated ASCs. This would enable the therapeutic cellular product to be instantly obtained with minimal contact with reagents, making it safer to use and associated with lesser regulatory criteria.

The SVF is defined as a heterogeneous population of freshly isolated cells from adipose tissue. The cell population comprises many different cell types, such as ASCs, lymphohematopoietic stem cells, endothelial cells, fibroblasts, and various immune cells, but excludes mature adipocytes [13, 14]. Several studies have shown improved results with SVF cells compared to ASCs. For example, freshly isolated SVF cells showed better results in promoting cartilage and subchondral bone regeneration [15]; and SVF was better than ASCs at forming new cartilage matrix when co-cultured with primary human chondrocytes [16]. However, we are unaware of any direct comparisons of SVF versus ASCs for treatment of denervated muscle.

In this study, we compared the secretomes of SVF and dASCs and found many factors that play various roles in muscle maintenance and regeneration that were more abundantly expressed in SVF cells compared with the dASCs. We then examined the effects of SVF on the proliferation and differentiation of myoblasts in a series of indirect co-culture experiments. Our data demonstrate how SVF cells enhanced the proliferation, but not differentiation, of myoblasts via secretion of HGF and activation of the MAPK/ERK pathway. These results provide new insight into the interaction of SVF cells and myoblasts and suggest that treatment with SVF preferentially leads to proliferation of myoblasts rather than differentiation into myotubes.

## Materials and methods

### Isolation of SVF cells

Cells from the stromal vascular fraction (SVF) were isolated from healthy female Sprague-Dawley rats (Taconic Europe A/S). The animal care and experimental procedures were carried out in accordance with the Directive 2010/63/EU of the European Parliament and of the Council on the protection of animals used for scientific purposes and was also approved by the Northern Swedish Committee for Ethics in Animal Experiments (No. A186-12). To isolate the cells, the fat was minced in 0.2% collagenase type I (Sigma-Aldrich; #C0130) in Hank’s Balanced Salt Solution (HBSS; Thermo Fisher Scientific; #14170088). The homogenate was placed in a 37 °C water bath for 60 min or until most of the fat had been digested. The enzyme solution was then neutralized using Minimum Essential Medium (MEM; Thermo Fisher Scientific; #32561029) supplemented with 10% fetal bovine serum (FBS; Thermo Fisher Scientific; #15140122), and centrifuged at 650×*g* for 5 min. After the stromal pellet had been re-suspended the solution was filtered through a 70-μm cell strainer and then centrifuged at 200×*g* for 5 min. Lastly, the red blood cells were lysed using Ammonium-Chloride-Potassium (ACK) lysing buffer (Thermo Fisher Scientific; #A1049201) for 3 min. The buffer was removed by centrifugation and the pellet was resuspended in MEM supplemented with 10% FBS and 1% penicillin-streptomycin. From this point and onwards, the cells were referred to as “SVF cells.” The cells were either used immediately or stored at − 80 °C for later use. A fraction of the cells were differentiated into a Schwann cell-like phenotype (dASCs), as described previously (4).

### Characterization of the SVF using flow cytometry

Flow cytometry was used to identify the proportions of the different cell types contained in the SVF, as previously described [14]. Cells (350000) were stained with anti-CD3 FITC (T cells; BD; #559975), anti-CD11b FITC (myeloid cells; BD; #561684), anti-CD45 PE/Cy7 (peripheral leukocytes; BD; #561588), anti-rat macrophage subset PE (BD; #554901), or anti-rat granulocytes FITC (BD; #554907) 30 min prior to analysis. Primary anti-CD36 (Abcam; #ab23680), secondary goat anti-mouse IgG (H + L) Alexa Fluor 594 (Thermo Fisher; #A-11005), and anti-CD45 PE/Cy7 (BD; #561588) antibodies were used to identify adipocytes. Primary anti-CD34 (R&D Systems; #AF6518-SP), secondary donkey anti-sheep IgG (H + L) PerCP (R&D Systems; #F0128), and anti-CD31 PE (BD; #555027) antibodies were used to identify lymphohematopoietic cells, endothelial cells, smooth muscle cells, and adipose-derived stem cells. Samples were run on a BD LSR II Flow Cytometer (BD). Ten thousand events were collected, and data was analyzed using the FACSDiva software (BD).

### Indirect co-culture of myoblasts and SVF cells

L6 rat myoblasts and C2C12 mouse myoblasts were cultured in Dulbecco’s Modified Eagle Medium (DMEM; Thermo Fisher Scientific; #31966021) containing l-alanyl-l-glutamine (GlutaMAX), 10% FBS, and 1% penicillin-streptomycin. Myoblasts were kept at a subconfluent level (< 80%) to prevent the loss of myoblastic component as the cells were passaged. In co-culture experiments where proliferation was studied, myoblasts were seeded in normal growth medium and then switched to low-serum medium (DMEM supplemented with 1% FBS) 24 h before exposing them to SVF cells. SVF cells were added at a 1:1, 1:2, or 1:5 ratio to myoblasts in 0.1 μm PET transwell membrane inserts (Corning; #353104) and co-cultured for up to 5 days. In pharmacological inhibitor experiments, MAP kinase kinase (MEK) inhibitor (25 μM; Calbiochem; #513001), Atropine (10^−5^ M, Sigma-Aldrich; #A0132), or Norleual (100 pM, Tocris; #5369) were added to cell cultures 2 h prior to adding the SVF cells. In cases where HGF (PeproTech; #100-39) was used the concentration varied from 5 to 30 ng/ml.

### BrdU proliferation assay

The proliferation rate of myoblasts was measured using Cell Proliferation Enzyme-Linked Immunoassay (Roche; #11647229001). Bromodeoxyuridine (BrdU) labeling solution was added to the culture wells to a final concentration of 10 μmol/L and incubated for 2 h at 37 °C. The cells were fixed for 30 min at room temperature and treated with anti-BrdU-peroxidase (POD) working solution for 60 min. The antibody conjugate was then removed and the cells were washed three times in phosphate-buffered-saline (PBS). For color development, substrate solution was added for 5 min. The absorbance was measured at 370 nm (reference wavelength 492 nm).

### Enzyme-linked immunosorbent assay (ELISA)

Secretion of hepatocyte growth factor (HGF) was measured using an ELISA according to the manufacturer’s instructions (RayBiotech; #ELR-HGF-1; detection range 0.8–200 ng/ml). Conditioned medium was collected from SVF cells and dASCs after 2 days of single culture and was analyzed immediately.

### Differentiation of myoblasts

L6 and C2C12 myoblasts were grown to near confluence in 6-well plates in normal growth medium for approximately 2 days (starting with 100,000 cells/well). The culture medium was then replaced with differentiation medium (DMEM containing 2% horse serum) to stimulate the formation of myotubes. At this point, cell culture inserts containing 250,000 SVF cells/insert were added to the culture wells. The cells were co-cultured for up to 7 or 14 days, with medium change every third day. During medium change, fresh SVF cells (250,000 cells/insert) were added to the culture inserts, as well as HGF, Norleual, and MEK inhibitor, in cases where that treatment was included. RNA and protein were extracted from myoblasts at day 7 and day 14.

### Immunocytochemistry

L6 and C2C12 myoblasts were seeded in 8 well culture slides (Falcon; #354108) in 250 μl normal growth medium. Once the cells had reached confluence the culture medium was replaced with differentiation medium. Cells were grown for 4 days after which they were washed three times with pre-warmed PBS with 0.1% BSA + 0.1% sodium azide; fixed with freshly pre-warmed 4% paraformaldehyde for 20 min in room temperature; washed again with PBS 3 × 5 min; blocked with 5% goat + 5% horse serum in PBS + 0.1% Triton X-100 for 15 min in room temperature; and stained for either fast type myosin heavy chain using mouse monoclonal antibody (1:40) (Leica Biosystems; #NCL-MHCf) or receptor c-Met using rabbit monoclonal antibody (1:100) (Abcam; #ab51067) for 2 h in room temperature. The cells were then washed with PBS 4 × 5 min; blocked once more with 5% goat + 5% horse serum in PBS; and stained with Alexa Fluor 488 goat anti-mouse secondary antibody (1:1000) (Invitrogen; #A11029) or Alexa Fluor 488 goat anti-rabbit secondary antibody (1:1000) (Invitrogen; #A11034). Slides were mounted with ProLong Diamond Antifade Mountant with DAPI (Invitrogen; #P36962) and examined in a Zeiss Axioskop 2 plus microscope equipped with an Olympus DP70 camera.

### Gene expression analysis using real-time quantitative PCR

Total RNA was extracted from cells using the RNeasy Mini Kit (Qiagen; #74106). cDNA was synthesized using the High Capacity cDNA Reverse Transcription kit (Applied Biosystems; #4268813), and qRT-PCR was performed with TaqMan gene expression assay (Applied Biosystems). Expression of 84 growth factors in SVF cells and dASCs was analyzed using the Rat Growth Factors RT^2^ PCR Array (Qiagen; #PARN-041ZC). The amplification was performed on a ViiA 7 Real-time PCR system (Applied Biosystems). Thermal-cycling conditions were 50 °C for 2 min, 95 °C for 20 s, and 40 cycles of 95 °C for 1 s, and 60 °C for 20 s. The expression of specific genes of interest was verified using the following probes: FIGF (Applied Biosystems; #Rn00582193), HGF (Applied Biosystems; #Rn00566673), and FGF10 (Applied Biosystems; #Rn00564115). Probes used for evaluating the degree of differentiation in myoblasts included: MYF5 (Applied Biosystems; #Rn01502778; #Mm00435125), MYH1 (Applied Biosystems; #Rn01751056; Mm01332489), and MYH2 (Applied Biosystems; #Rn01470656; Mm01332564). Data were analyzed with ViiA 7 software (Applied Biosystems) and PCR Array Analysis Web Portal (Qiagen). The expression was normalized to Rpl13a levels (Applied Biosystems; #Rn00821946).

### Western blotting

Myoblasts were lysed in RIPA buffer supplemented with protease inhibitor (Sigma-Aldrich), and total protein was quantified using BioRad Protein Assay (Bio-Rad Laboratories; #500-0006). Samples were run on pre-cast polyacrylamide gels (Bio-Rad Laboratories) for 60 min at 150 V. The gels were transferred to polyvinylidene fluoride (PVDF) membranes and run for 60 min at 100 V. Membranes were blocked in 5% bovine serum albumin (BSA; Sigma-Aldrich; #A2058) in TBST (10 mM Tris Base, 100 nM NaCl, 0.1% Tween-20) for 60 min at room temperature and stained with antibodies against p-ERK1/2 (1:2000) (Cell Signaling; #4370), ERK1/2 (1:1000) (Cell Signaling; #4695), fast type MyHC (1:500) (Leica Biosystems; #NCL-MHCf), or β-actin (1:2000) (Cell Signaling; #4970) over night at 4 °C. Membranes were washed with TBST for 6 × 5 min, after which HRP-conjugated secondary antibodies (1:2000) (Cell Signaling; #7074 or #7076) were added for 60 min at room temperature. Finally, membranes were incubated in ECL solution (GE Healthcare; #RPN2232) for 60 s and analyzed in an Odyssey Fc Dual-Mode Imaging System (LI-COR Biosciences).

### Statistical analysis

All experiments were conducted at least three times using cell preparations obtained from at least three different rats. Results are presented as mean ± standard deviation. Statistical analysis was performed using GraphPad Prism 7. Statistical differences were analyzed using one-way analysis of variance (ANOVA) with post hoc test (Bonferroni correction) or Student’s *t*-test. *P* < 0.05 was considered statistically significant.

## Results

### Adipose tissue SVF contains a mixture of stem cells and immune cells

SVF cells were characterized using flow cytometry as described by Bowles et al. [14]. Our results confirmed that the stromal vascular fraction contained a mixture of regenerative cell types and various immune cells, but excluded mature adipocytes (Table 1).

**Table 1:** Characterization of the stromal vascular fraction (SVF) cells using flow cytometry (*n* = 3 animals)

Designated cell type	Cell marker expression	Mean (%) ± SD
Adipocytes	CD45^−^ CD36^+^	0.4 ± 0.22
Adipose-derived stem cells	CD34^+^ CD31^−^	31.53 ± 14.81
Endothelial cells	CD34^+^ CD31^+^	2.77 ± 2.83
Smooth muscle cells	CD34^dim^ CD31^−^	6.05 ± 1.65
Lymphohematopoietic cells	CD34^+^	43.67 ± 4.30
Macrophages	Anti-Rat Macrophage Subset	34.83 ± 5.25
Granulocytes	Anti-Rat Granulocytes	19.27 ± 3.90
Myeloid cells	CD11b^+^	30.47 ± 3.12
T cells	CD3^+^	41.23 ± 7.10
Peripheral leukocytes	CD45^+^	67.60 ± 7.15

### SVF cells enhance the proliferation of myoblasts through secretion of growth factors

**Fig. 1:**
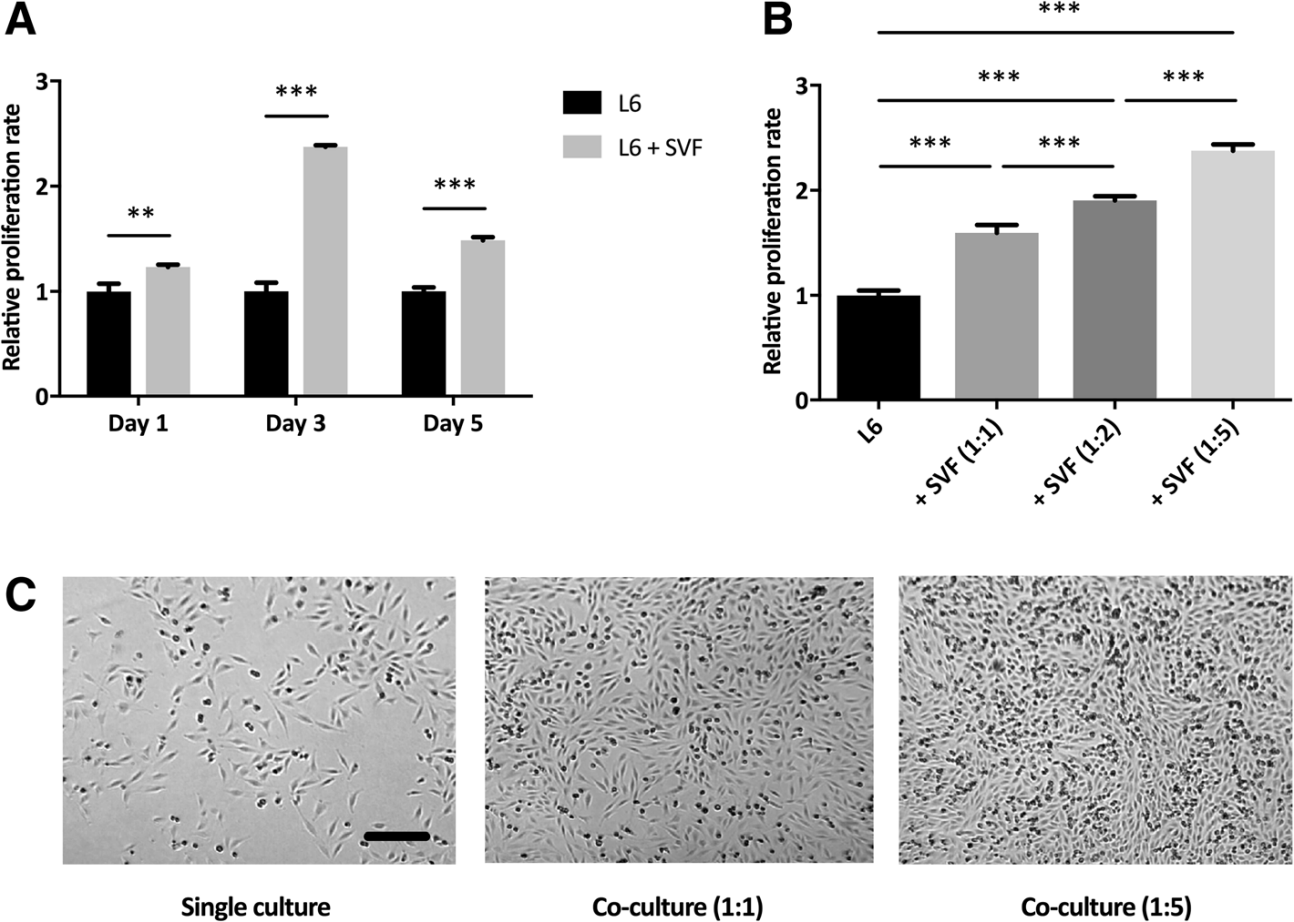
SVF cells enhance the proliferation of L6 myoblasts. **a** Indirect co-culture for 1, 3, and 5 days, compared to myoblasts cultured alone. SVF cells were added at a ratio of 1:5 to myoblasts. The proliferation rate was measured using BrdU proliferation assay. Statistical analysis was performed using the Student *t* test. **b** Proliferation rate after 3 days of co-culture using three different concentrations of SVF cells. Statistical analysis was performed using one-way ANOVA with post hoc test (Bonferroni correction). **c** Visualization of the L6 myoblasts after 3 days of co-culture using different concentrations of SVF. Bar = 200 μm. Results are presented as mean ± standard deviation. *n* = 3. **P < 0.01 and ***P < 0.001

Myoblasts co-cultured with SVF cells had an increased proliferation rate compared to myoblasts cultured alone (Fig. 1a). The proliferation rate was significantly enhanced for all measured time points (1, 3, and 5 days). Furthermore, the response was SVF dose-dependent (Fig. 1b). The increased number of cells was readily apparent when cultures were viewed under a light microscope (Fig. 1c).

### SVF cells enhance the proliferation of myoblasts but not via an acetylcholine-dependent mechanism

**Fig. 2:**
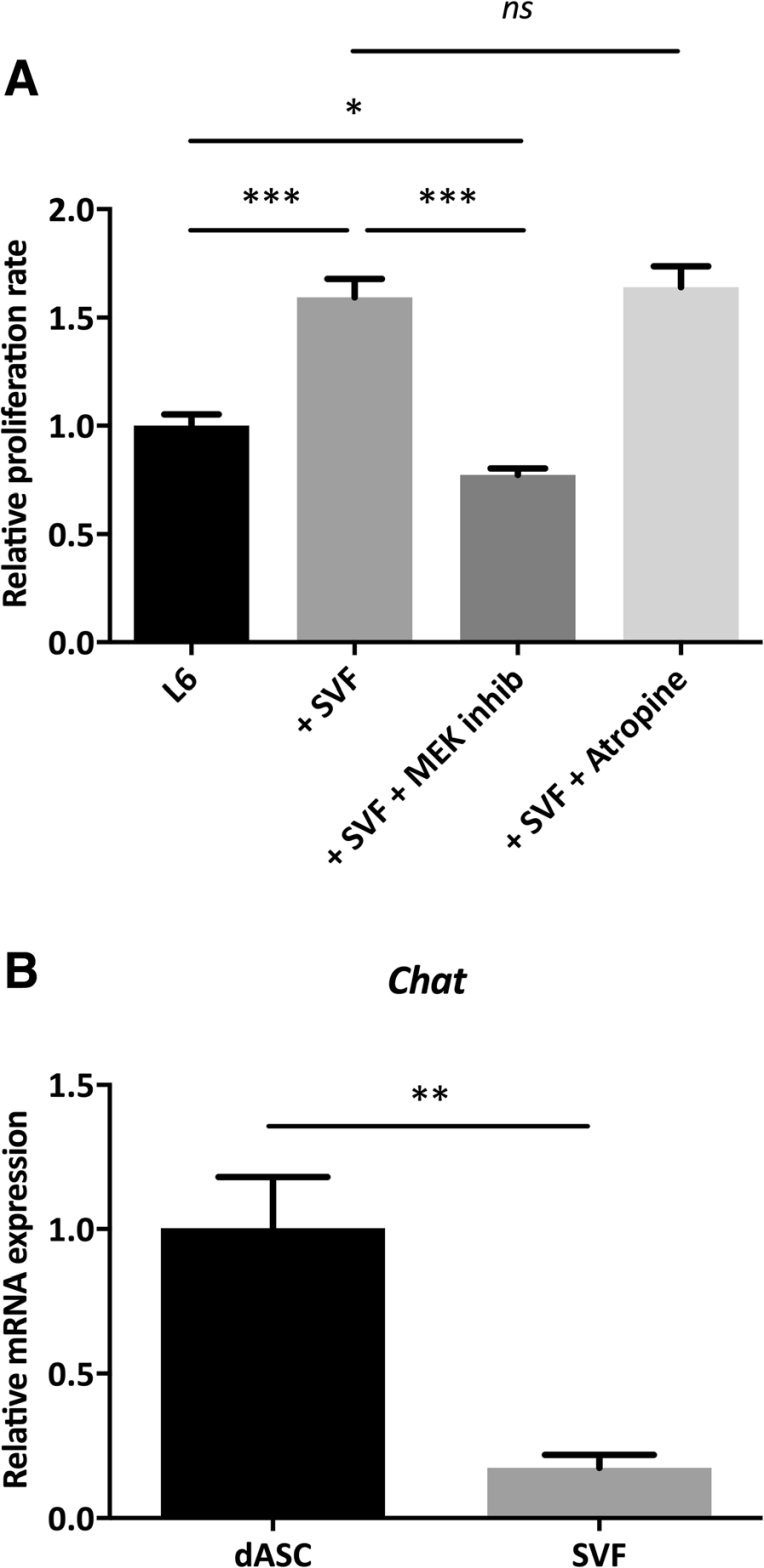
SVF cells enhance the proliferation of L6 myoblasts via the MAPK/ERK pathway. **a** Indirect co-culture for 3 days in the presence of a MEK inhibitor (inhibits the phosphorylation of MAP kinase substrates, such as ERK) or atropine (antagonist of the muscarinic acetylcholine receptors). Statistical analysis was performed using one-way ANOVA with post hoc test (Bonferroni correction). **b** mRNA expression of choline acetyltransferase (ChAT) in differentiated adipose stem cells (dASC) and SVF cells. Statistical analysis was performed using the Student *t* test. Results are presented as mean ± standard deviation. *n* = 3. *P < 0.05, **P < 0.01, and ***P < 0.001. *ns*, not significant

The enhanced proliferation of myoblasts in the coculture experiments was blocked in the presence of a MAP kinase kinase (MEK) inhibitor, which suggested that the effect was mediated via the MAPK/ERK pathway (Fig. 2a). Since we have shown previously that dASCs enhanced myoblast proliferation through secretion of acetylcholine (ACh), we included atropine, a muscarinic acetylcholine (ACh) receptor antagonist, in our co-cultures. This did not have an effect on the proliferation rate (Fig. 2a). Furthermore, compared with the dASCs, the SVF cells expressed significantly lower levels of choline acetyltransferase (ChAT), an enzyme which is necessary for ACh production (Fig. 2b). Thus, SVF cells and dASCs do not share the same mechanism to increase the proliferation of myoblasts.

### SVF cells express and secrete HGF at concentrations sufficient to enhance the proliferation of myoblasts

**Fig. 3:**
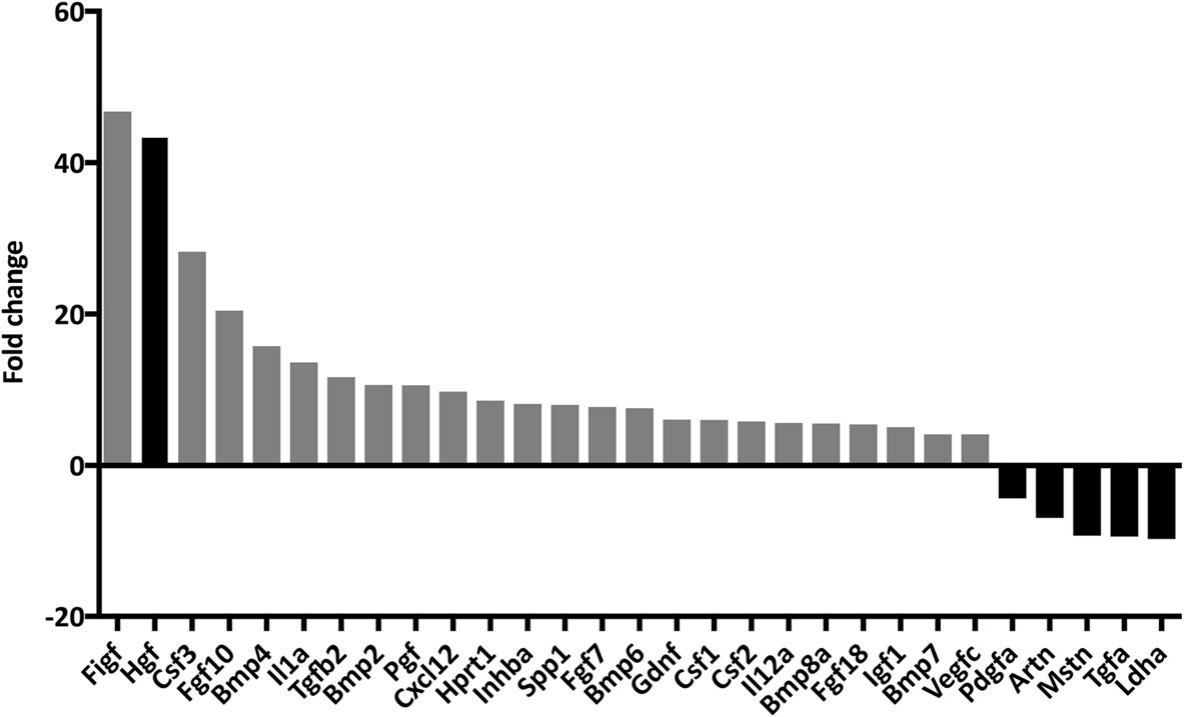
SVF cells have an increased expression of a multitude of growth factors compared with differentiated adipose stem cells (dASCs), including hepatocyte growth factor (HGF). Bars indicate the mRNA expression in SVF cells compared with the expression in dASCs

In SVF cells, a large number of growth factors had an increased expression compared with dASCs (Fig. 3a). Genes of particular interest included c-Fos-induced growth factor (FIGF; 47-fold increase), hepatocyte growth factor (HGF; 43-fold increase), and fibroblast growth factor 10 (FGF10; 20-fold increase). The elevated expression of these genes was verified using real-time qPCR (data not shown). We subsequently focused on HGF because of its well-documented effects in muscle [17]. In conditioned media from SVF cells, a concentration range of 2.2–13.6 ng/ml HGF protein was confirmed using ELISA (Table 2). No HGF was detected in media from dASCs.

**Table 2:** HGF in media measured by ELISA

Cell population	HGF in media
dASC (*n* = 3 animals)	Not detectable
SVF (*n* = 4 animals)	2.2–13.6 ng/ml

### Blocking of the c-Met receptor significantly reduces the SVF-induced proliferation of myoblasts

**Fig. 4:**
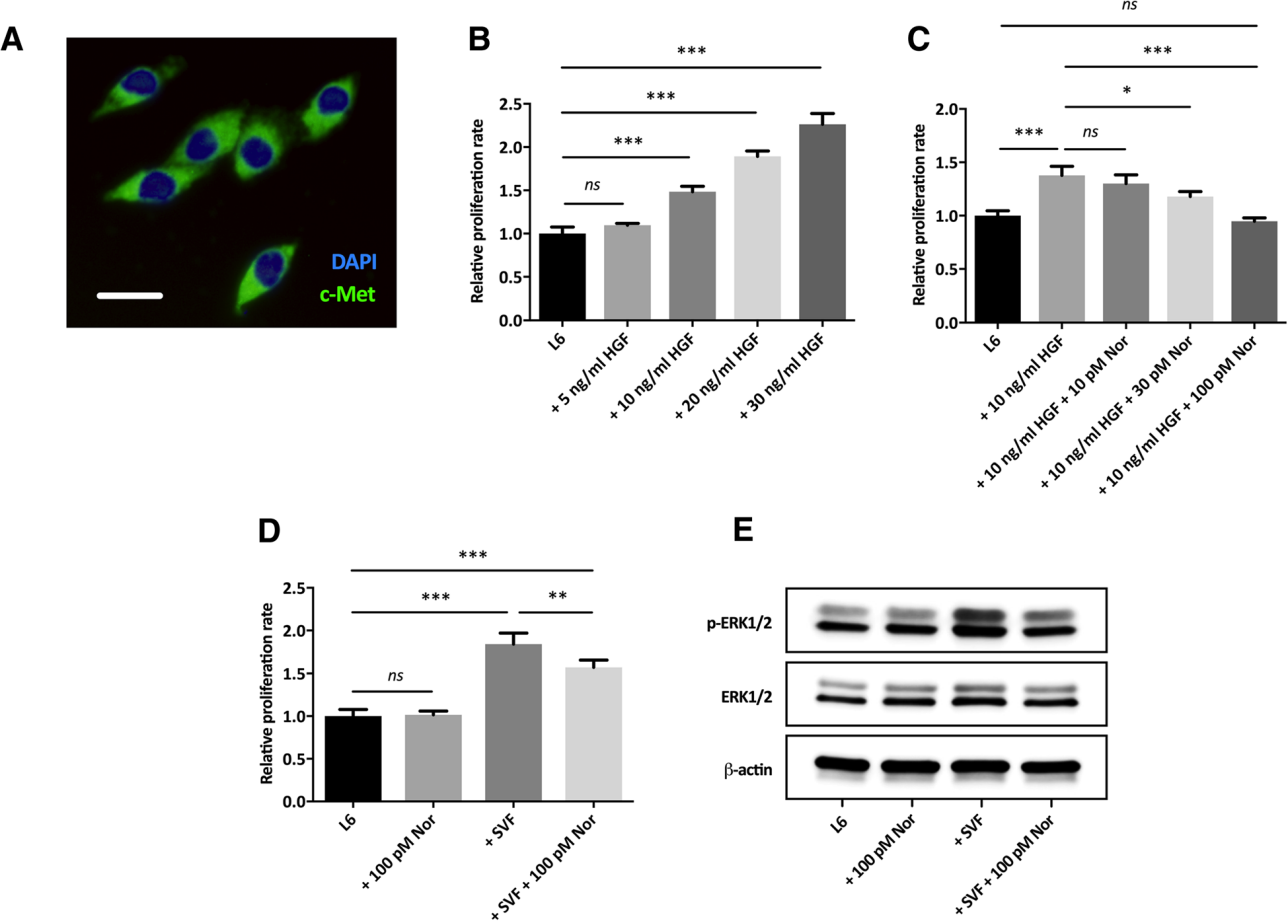
The SVF-induced proliferation of L6 myoblasts is reduced by Norleual (Nor), an HGF inhibitor. **a** Immunofluorescence staining of the HGF receptor c-Met (green) in L6 myoblasts, counterstained with DAPI (blue). Bar = 10 μm. **b** The effect of different concentrations of HGF on the proliferation rate of L6 myoblasts. **c** 100 pM Norleual effectively inhibits the proliferation induced by 10 ng/ml HGF. **d** Indirect co-culture of L6 myoblasts and SVF cells for 3 days in the presence of 100 pM Norleual. Norleual was tested on cells alone and no toxicity could be observed. **e** The expression of p-ERK1/2 and total ERK1/2 protein in L6 myoblasts after co-culture with SVF cells. Norleual had no effect on baseline levels of p-ERK1/2. Statistical analysis was performed using one-way ANOVA with post hoc test (Bonferroni correction). Results are presented as mean ± standard deviation. *n* = 3. *P < 0.05, **P < 0.01, and ***P < 0.001. *ns*, not significant

The expression of the HGF receptor c-Met was confirmed in L6 myoblasts using real-time qPCR (CT value of 23) and immunocytochemistry (Fig. 4a). Exogenous HGF (10–30 ng/ml) significantly increased the proliferation rate of myoblasts (Fig. 4b). Inclusion of 100 pM Norleual, a HGF inhibitor, effectively blocked the proliferation induced by 10 ng/ml HGF (Fig. 4c). SVF cells and myoblasts were co-cultured in the presence of 100 pM Norleual, which significantly reduced the SVF-induced proliferation of myoblasts (Fig. 4d). Western blotting revealed that p-ERK1/2 was upregulated in myoblasts after co-culture with SVF cells and that this was due to binding of HGF to its receptor, since the addition of Norleual reduced the expression to baseline levels (Fig. 4e).

### SVF cells inhibit the differentiation of myoblasts into mature myotubes

**Fig. 5:**
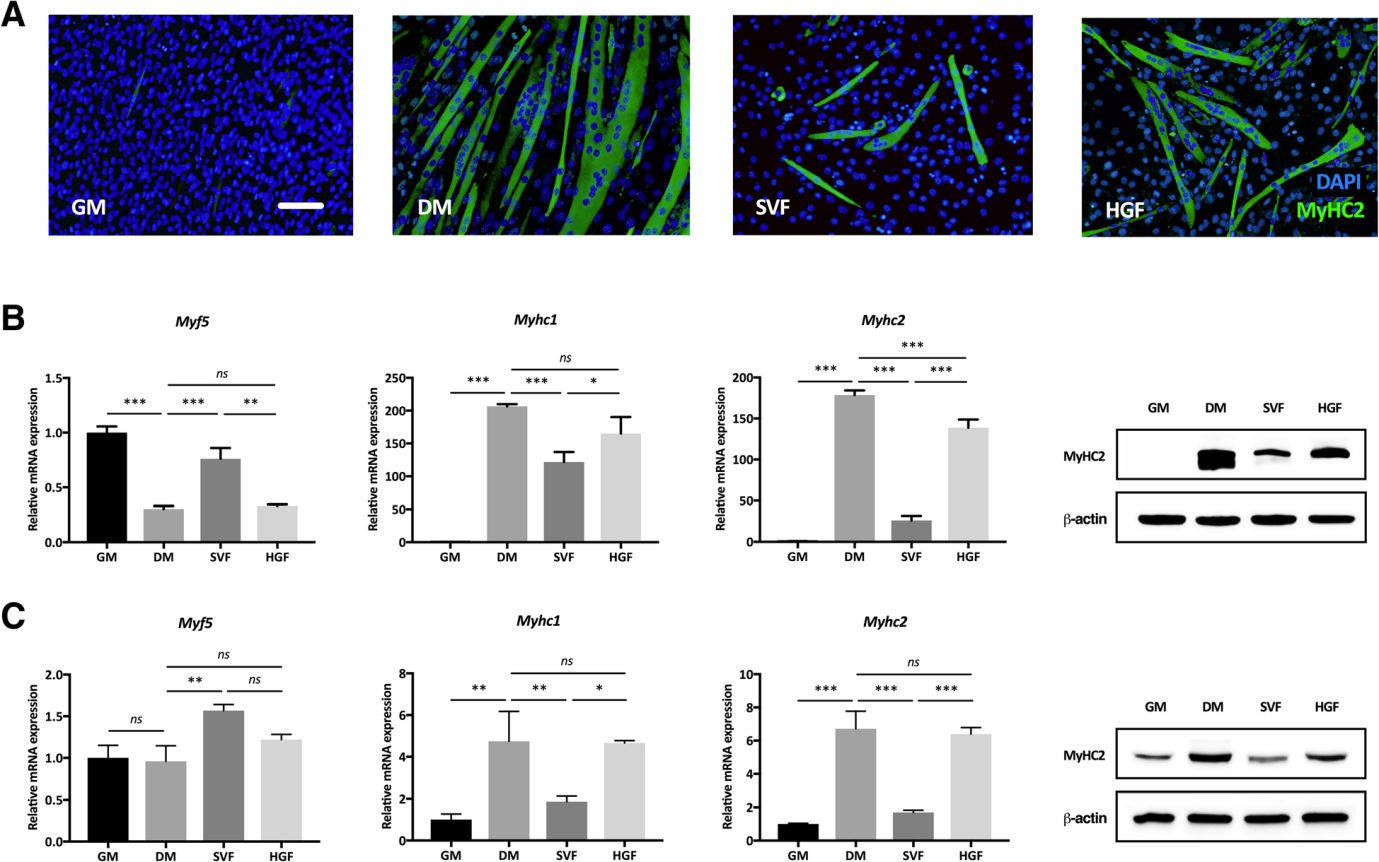
Differentiation of myoblasts into myotubes is inhibited by SVF cells and HGF. **a** Immunofluorescence staining of fast type myosin heavy chain (green) in C2C12 mouse myoblasts after 4 days in growth medium (GM), differentiation medium (DM), indirect co-culture with SVF cells in DM (SVF), and DM supplemented with 10 ng/ml HGF (HGF). Cells were counterstained with DAPI (blue). Bar = 200 μm. The expression of myogenic regulatory factor 5 (*Myf5*), slow type myosin heavy chain (*Myhc1*), and fast type myosin heavy chain (*Myhc2*) mRNA in C2C12 myoblasts after **b** 7 days or **c** 14 days of differentiation in the various culture conditions. Western blot of fast type myosin heavy chain (MyHC2) protein is also shown. β-actin was used as a loading control. Statistical analysis was performed using one-way ANOVA with post hoc test (Bonferroni correction). Results are presented as mean ± standard deviation. *n* = 3. *P < 0.05, **P < 0.01, and ***P < 0.001. *ns*, not significant

We found that the L6 rat myoblasts did not efficiently differentiate into myotubes (see Additional file 1: Figure S1), so we decided to switch to the more commonly used C2C12 cell line. Already after 4 days in differentiation medium (DM), multinucleated tube-like structures, which stained positively for fast type myosin heavy chain protein (MyHC2) (Fig. 5a), were observed. The C2C12 myoblasts cultured in the presence of SVF cells displayed markedly fewer myotubes as compared to DM alone. The number of myotubes formed in DM supplemented with HGF was also reduced but not to the same extent as in the presence of SVF (Fig. 5a). Similar observations were made at later time points, and this correlated with lower levels of myosin heavy chain isoform 1 (*Myhc1*) and 2 (*Myhc2*) mRNA in the presence of SVF after 7 days (Fig. 5b) and 14 days (Fig. 5c) of differentiation. The inhibitory effect of SVF cells on MyHC2 protein was confirmed by Western blotting (Fig. 5b, c). The mRNA expression of myogenic regulatory factor 5 (*Myf5*), one of the earliest markers of myogenesis [18], was inversely related to the myotube formation. The addition of Norleual, an HGF inhibitor, or MEK inhibitor to the C2C12 + SVF co-cultures made no significant difference on the expression of fast myosin heavy chain mRNA or protein as compared to C2C12 + SVF alone (see Additional file 2: Figure S2).

## Discussion

We have previously reported that Schwann cell-like differentiated adipose-derived stem cells (dASCs) enhanced the proliferation of myoblasts through paracrine secretion of acetylcholine (ACh) [10]. In this study, we found that SVF cells are also potent in inducing proliferation of myoblasts in cell culture but not via an acetylcholine-dependent mechanism. In contrast to dASCs, SVF cells did not express choline acetyltransferase (Chat), nor did atropine, a muscarinic ACh receptor antagonist, have an effect on the proliferation rate. To enlighten some of the differences between the SVF and dASC secretomes we performed a Growth Factor PCR Array, which led to the finding that HGF is one of the highest expressed growth factors in SVF cells among the growth factors that were tested. We confirmed the high expression by running qPCR on samples from several rats, all with similar results. This finding was of particular interest since HGF is known to exert organo-protective and regenerative features [17]. For example, HGF stimulates cell proliferation, motility, morphogenesis, and angiogenesis and is required for self-repair after injuries to the skin, muscle, and cartilage [19–21], while HGF neutralization or c-Met gene knockout leads to enhanced apoptosis, inhibition of cell proliferation, and retarded repair. In one study, when anti-HGF IgG was injected into mice during muscular damage, myoblast proliferation and muscular fiber formation were suppressed [22]. It is known that within days after denervation, there is an activation of the myogenic program in denervated muscle; satellite cells become activated and there is an increase of myogenic regulatory factors [23–25]. This is a sign of self-repair, but the window is only open for approximately 2 months before satellite cell numbers start to fall below control levels [26, 27]. It has also been reported that during muscular damage, HGF is secreted by satellite cells in an autocrine fashion [28]. This makes it tempting to speculate that intramuscular SVF injections, with the aim of preventing muscle atrophy, could boost the HGF-c-Met self-repair system, and if given at an early stage, might also prolong the life span of the satellite cell population. Another interesting feature of HGF is that it inhibits TGF-β production in cultures of myofibroblasts [29, 30]. Since TGF-β plays an important role in tissue fibrosis by converting fibroblasts to ECM-producing myofibroblasts [20, 21], and long-term denervated muscle is characterized by overly active myofibroblasts and interstitial fibrosis [31], SVF cells might very well have anti-fibrotic outcomes in vivo.

In our study, when exogenous HGF was administered, a concentration of 10 ng/ml HGF was needed to induce cell proliferation. We performed an ELISA to determine the amount of HGF secreted in our co-cultures and found that the concentration varied from 2.2–13.6 ng/ml. Although the concentration of HGF in cultured SVF cells was not in all cases as high as 10 ng/ml, one has to bear in mind that it is continuously released over time. As for the downstream response to c-Met activation, it varies depending on adaptor molecules: recruitment of the GRB2-SOS complex activates the Ras-Raf-MEK-ERK pathway [32, 33] leading to cell proliferation and cell motility, while phosphorylation of GAB1, a docking protein that couples c-Met to more signaling effectors such as PI3K [34, 35], leads to morphogenesis and cell survival. In our study, the activation of the MAPK/ERK pathway in myoblasts after co-culture with SVF cells was confirmed by Western blotting. When a c-Met inhibitor was included, the proliferation rate was reduced, however not to the same extent as when a MEK inhibitor was included in the experiment. This suggests that SVF cells exert their proliferative effect via the MAPK/ERK pathway but does not depend on HGF secretion alone. Instead, it is likely that multiple growth factors work together to reach the proliferative effect. Such growth factors could be FGF and FIGF, both of which can lead to ERK phosphorylation [36, 37], and which we found to be more highly expressed in SVF cells in comparison with dASCs.

In contrast to the results of the proliferation studies we found that SVF cells inhibited the formation of myotubes and reduced the expression levels of myosin heavy chain genes and proteins in a model system of myogenic differentiation. Already after 4 days there were clear differences in the staining pattern for fast type myosin heavy chain (MyHC2) between the DM-treated group and the SVF-treated group. Interestingly, HGF administration had a similar effect, and the effect was significant for up to 7 days. This is rather surprising considering the important function of HGF in damaged muscle, but HGF is in fact known to play a dual role in myoblast proliferation and differentiation, effects that are dose dependent [38]. HGF stimulates proliferation and cell-renewal, but inhibits differentiation by inhibiting the heterodimerization process of myogenic regulatory factors [39]. It should however be noted that the addition of Norleual, an HGF inhibitor, did not change the level of differentiation in the SVF-treated group, which means that HGF is not a key player in the effects on differentiation. There are likely other factors secreted by the SVF that contribute to the inhibitory effects on differentiation. These results could also be explained by interactions among the growth factors in the SVF cell secretome, i.e. alone HGF might have an effect but in combination with other growth factors the effect might be canceled out. Nonetheless, SVF cells reduced the differentiation of myoblasts in vitro. This is consistent with the fact that *Myf5*, one of the earliest markers of myogenesis [18], is upregulated in myoblasts co-cultured with SVF cells, i.e., myoblasts are kept at a premature stage when exposed to the SVF. Based on our findings, we argue that the SVF is a potent proliferative agent that could be used to preserve denervated muscle in vivo, but its role in neomyogenesis needs further investigation.

In summary, it can be speculated that SVF cells harbor stimulatory effects that are contradictory: either preserving the denervated muscle by increasing the proliferation of myoblasts, including satellite cells, or by inhibiting myotube formation and thus preserving cells in a more immature state. Furthermore, it might be that once a muscle is re-innervated after denervation, the myoblasts could differentiate as a response to regained mechanical loading.

## Conclusion

Stem cells represent a new paradigm in medicine, and we believe that SVF cells could be used to preserve denervated muscle until the nerve supply has been fully restored, if not for neomyogenesis then for muscle mass maintenance. Various mechanisms could be involved, including proliferation of myoblasts through secretion of growth factors such as HGF; activation of satellite cells; direct cell-cell interactions; and in vivo differentiation of transplanted cells. However, more research is needed to fully understand the mechanism of action, but given that SVF cells secrete such a vast array of growth factors, we predict that intricate paracrine activity could play an important role in vivo.

## Additional files

Additional file 1: **Figure S1.** L6 myoblasts showed poor ability to differentiate into multinucleated myotubes. (A) Immunofluorescence staining of fast type myosin heavy chain (green) after 7 days in growth medium (GM) and differentiation medium (DM). Cells were counterstained with DAPI (blue). No myotubes could be observed. Bar = 200 μm. (B) The expression of *Myf5*, *Myhc1*, and *Myhc2* mRNA in L6 myoblasts after 7 days of differentiation in different culturing conditions. Statistical analysis was performed using one-way ANOVA with post hoc test (Bonferroni correction). Results are presented as mean ± standard deviation. *n* = 3. *P < 0.05, **P < 0.01, and ***P < 0.001. *ns*, not significant. (TIFF 7008 kb)

Additional file 2: **Figure S2.** The addition of Norleual and MEK inhibitor did not alter the inhibitory effect of SVF cells on the differentiation of C2C12 myoblasts. (A) The expression of *Myhc2* mRNA after 14 days in growth medium (GM); differentiation medium (DM); indirect co-culture with SVF cells in DM (SVF); indirect co-culture with SVF cells in DM supplemented with 100 pM Norleual (SVF + N); and indirect co-culture with SVF cells in DM supplemented with 25 μM MEK inhibitor (SVF + M). (B) The expression of MyHC2 protein in C2C12 cells after 14 days of differentiation. Statistical analysis was performed using one-way ANOVA with post hoc test (Bonferroni correction). Results are presented as mean ± standard deviation. *n* = 3. ***P < 0.001. *ns*, not significant. (TIFF 553 kb)

## Abbreviations

ACh: Acetylcholine
ASC: Adipose stem cell
BrdU: Bromodeoxyuridine
ChAT: Choline acetyltransferase
dASC: Differentiated adipose stem cell
ERK: Extracellular signal-regulated kinase
HGF: Hepatocyte growth factor
MEK: MAP kinase kinase
MSC: Mesenchymal stem cell
MyHC: Myosin heavy chain
qPCR: Quantitative polymerase chain reaction
SVF: Stromal vascular fraction
