# Supplementary figures and images for: The adipose tissue stromal vascular fraction secretome enhances the proliferation but inhibits the differentiation of myoblasts
Source: Stem Cell Res Ther. 2018 Dec 20;9:352. doi: 10.1186/s13287-018-1096-6 (PMC6302486; doi:10.1186/s13287-018-1096-6)

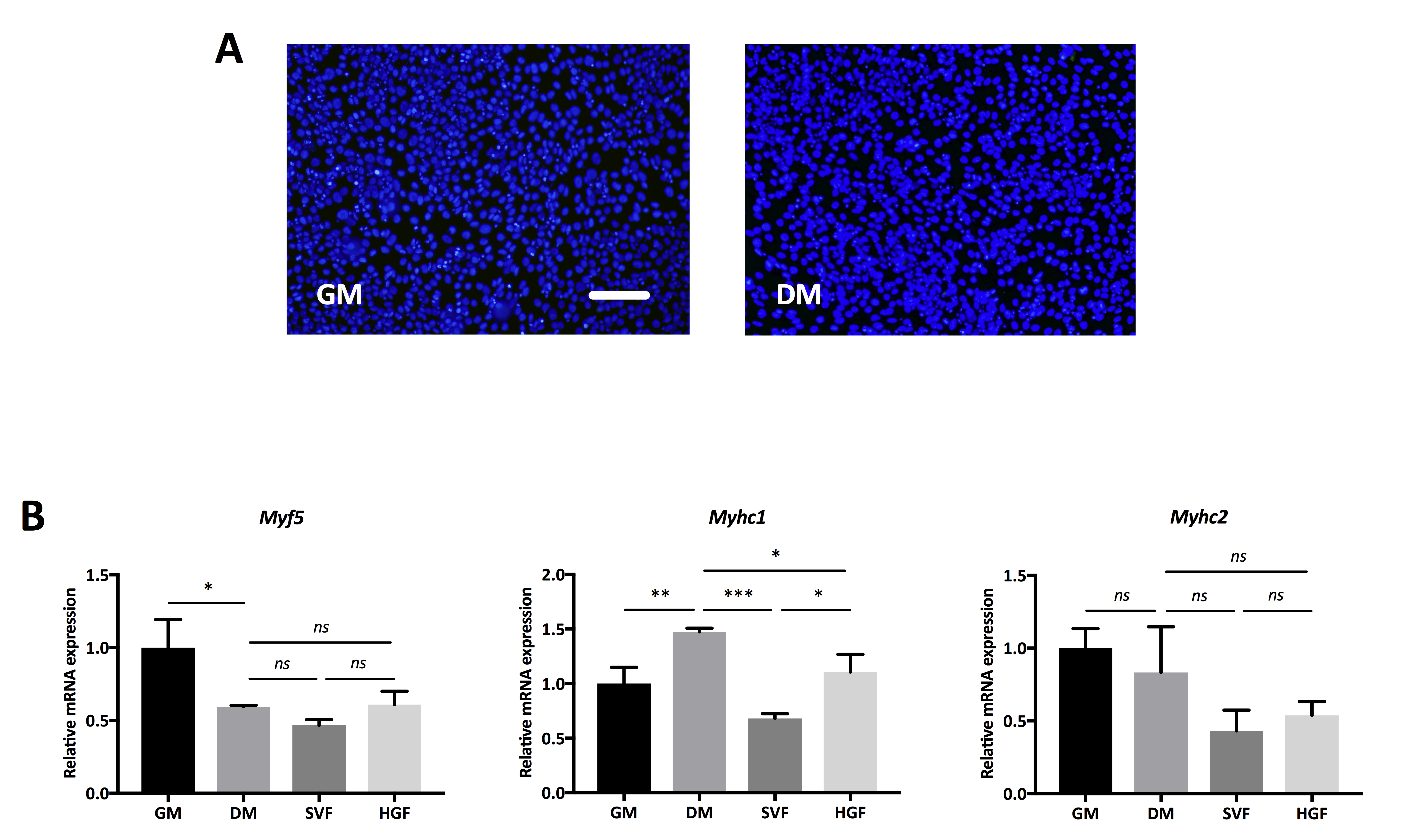

Supplement: Supplementary file 1 — Figure S1. L6 myoblasts showed poor ability to differentiate into multinucleated myotubes. (A) Immunofluorescence staining of fast type myosin heavy chain (green) after 7 days in growth medium (GM) and differentiation medium (DM). Cells were counterstained with DAPI (blue). No myotubes could be observed. Bar = 200 μm. (B) The expression of Myf5, Myhc1, and Myhc2 mRNA in L6 myoblasts after 7 days of differentiation in different culturing conditions. Statistical analysis was performed using one-way ANOVA with post hoc test (Bonferroni correction). Results are presented as mean ± standard deviation. n = 3. *P < 0.05, **P < 0.01, and ***P < 0.001. ns, not significant. (TIFF 7008 kb) [file 13287_2018_1096_MOESM1_ESM.tiff]

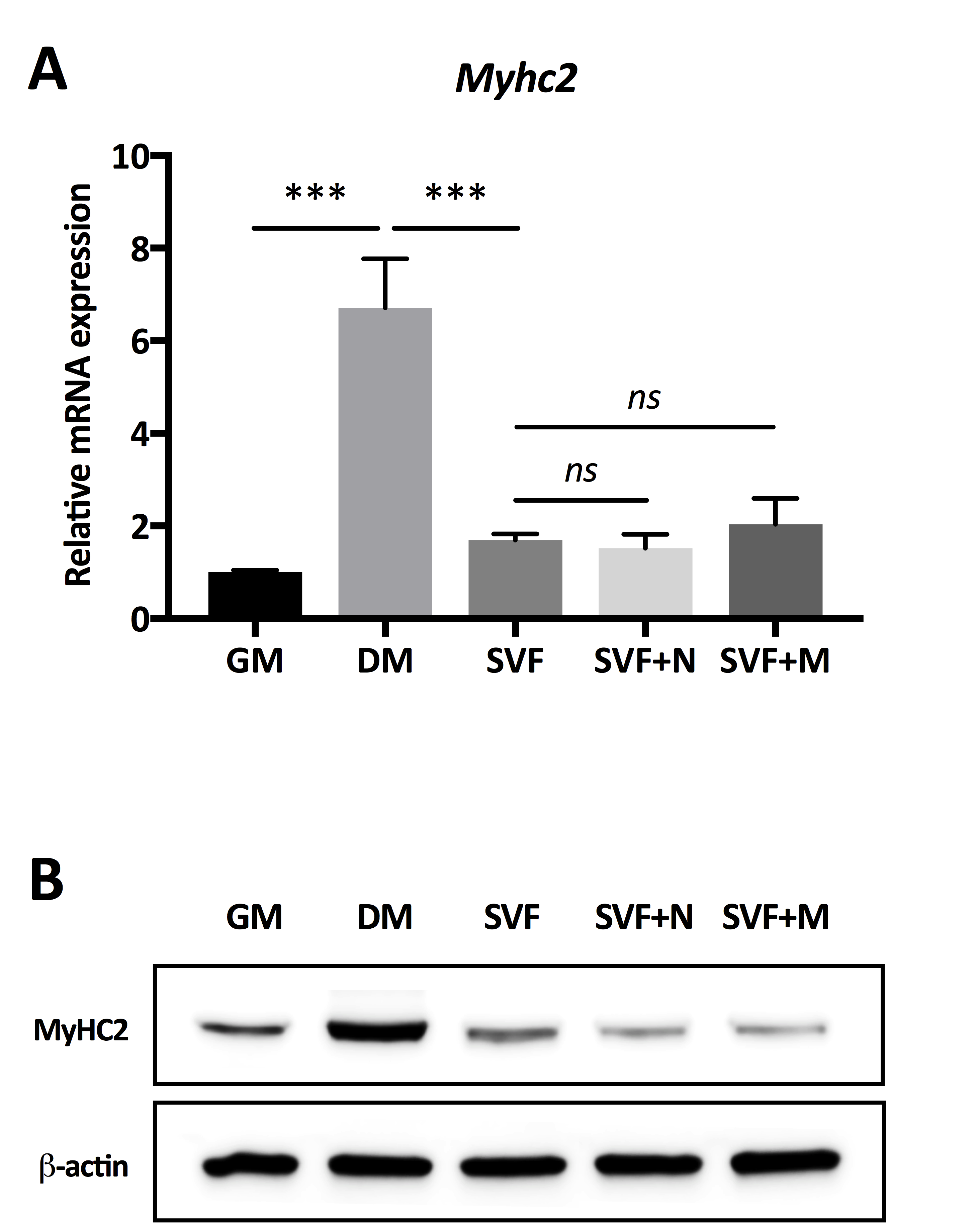

Supplement: Supplementary file 2 — Figure S2. The addition of Norleual and MEK inhibitor did not alter the inhibitory effect of SVF cells on the differentiation of C2C12 myoblasts. (A) The expression of Myhc2 mRNA after 14 days in growth medium (GM); differentiation medium (DM); indirect co-culture with SVF cells in DM (SVF); indirect co-culture with SVF cells in DM supplemented with 100 pM Norleual (SVF + N); and indirect co-culture with SVF cells in DM supplemented with 25 μM MEK inhibitor (SVF + M). (B) The expression of MyHC2 protein in C2C12 cells after 14 days of differentiation. Statistical analysis was performed using one-way ANOVA with post hoc test (Bonferroni correction). Results are presented as mean ± standard deviation. n = 3. ***P < 0.001. ns, not significant. (TIFF 553 kb) [file 13287_2018_1096_MOESM2_ESM.tiff]
